# High Performance Ultrathin MoO_3_/Ag Transparent Electrode and Its Application in Semitransparent Organic Solar Cells

**DOI:** 10.3390/nano8070473

**Published:** 2018-06-27

**Authors:** Linlin Shi, Yanxia Cui, Yupeng Gao, Wenyan Wang, Ye Zhang, Furong Zhu, Yuying Hao

**Affiliations:** 1 Key Lab of Advanced Transducers and Intelligent Control System of Ministry of Education, College of Physics and Optoelectronics, Taiyuan University of Technology, Taiyuan 030024, China; shilinlinsll@sina.cn (L.S.); gaoyupeng@boe.com.cn (Y.G.); wangwenyan@tyut.edu.cn (W.W.); zhangye@tyut.edu.cn (Y.Z.); frzhu@hkbu.edu.hk (F.Z.); haoyuyinghyy@sina.com (Y.H.); 2 Department of Physics and Institute of Advanced Materials, Hong Kong Baptist University, Kowloon 22100, Hong Kong, China

**Keywords:** transparent electrode, Ag film, nucleation layer, organic solar cell

## Abstract

In this paper, we demonstrate high performance ultrathin silver (Ag) transparent electrodes with a thin MoO_3_ nucleation layer based on the thermal evaporation method. The MoO_3_/Ag transparent electrodes fabricated at different deposition rates were compared systematically on aspects of the transmission spectrum, surface resistance, and surface morphology. Our study indicates that with the presence of the MoO_3_ nucleation layer, an Ag film of only 7 nm thick can achieve percolation and the film is porous instead of forming isolated islands. In addition, the increase of the deposition rate can yield obvious improvement of the surface morphology of the Ag film. Specifically, with the help of a 1 nm thick MoO_3_ nucleation layer, the Ag film of 9 nm thick realized under the deposition rate of 0.7 nm/s has a surface resistance of about 20 ohm/sq and an average transmittance in the visible light range reaching 74.22%. Such a high performance of transmittance is superior to the reported results in the literature, which inevitably suffer obvious drop in the long wavelength range. Next, we applied the ultrathin MoO_3_/Ag transparent electrode in organic solar cells. The optimized semitransparent organic solar cell displays a power conversion efficiency of 2.76% and an average transmittance in the visible range of 38% when light is incident from the Ag electrode side.

## 1. Introduction

Organic solar cells (OSCs), showing advantages in terms of rich material resources, light weight, good compatibility with roll-to-roll and large area fabrications, colorfulness, etc., have become a very hot research topic in the field of solar energy utilization [1]. In OSCs, Indium Tin Oxide (ITO) is the most common transparent electrode. However, the ITO transparent electrodes suffer a series of limitations due to their poor mechanical flexibility, high cost, and the rareness of indium [2]. To replace ITO, other transparent conductive materials with high transmittance, low surface resistance, good flexibility, and low cost are being developed. Different materials have been proposed to replace ITO as transparent electrodes, e.g., highly conductive polymers (e.g., Poly(3,4-ethylenedioxythiophene): poly(styrenesulfonate), shorted as PEDOT:PSS) [3], metal nanostructures [4,5,6,7,8,9,10,11,12], graphene [13,14], carbon nanotubes [15], etc. Among these conductive materials, noble metals including silver (Ag) and gold (Au), which have high conductivity and are inert from oxidation, have been frequently used to make transparent electrodes. Compared to Au, Ag has relatively lower resistivity [16]; besides, Ag is more inexpensive than Au. Therefore, Ag based transparent electrodes have attracted intensive attention in the field of optoelectronics [16,17,18].

Ag nanowires show high transmission and electrical conductivity [17], but they can produce interference and scattering effects in the range of visible light. Besides, it is not easy to attach them onto the photosensitive layer, resulting in poor performance of the device and other issues [18,19]. In contrast, a single layer of Ag film which also shows good conductivity does not generate any negative optical effects to affect the device performance. However, an Ag film with a preferable surface resistance has a large thickness due to its poor wettability on insulating substrates such as silicon dioxide, thereby exhibiting low transmittance [20,21]. Doping the Ag film with a small amount of calcium or aluminum is an effective method to greatly reduce the percolation thickness to form a continuous film, resulting in high transmittance at the same time with a preferable surface resistance [22,23]. However, the doping process in the experiment can hardly be controlled in obtaining a fixed ratio of the alloy film, especially when the thermal evaporation method is used. 

Researchers have also attempted to realize thin Ag film by introducing a nucleation layer which can facilitate Ag percolation. Metals, like nickel [24], germanium [25,26], chromium [27], etc., have been frequently used as the nucleation layer for Ag growth, but these high glossy metals bring about unavoidable optical loss and they also alter the plasmonic properties of the Ag films. Metal oxides are another great choice of the nucleation layer for Ag growth. Some poor transparent conductive oxides, e.g., ZnO [28,29], TiO_2_ [30,31], SnO_x_ [32,33], Nb_2_O_5_ [34], and so on, have been researched frequently for this purpose. However, their depositions require the magnetic sputtering method or electron/ion-beam assisted techniques, causing possible damage of the organic films. In this regard, transition metal oxides like MoO_3_ [35,36,37], WO_3_ [38,39,40], and V_2_O_5_ [41] produced by the thermal evaporation method, have been proposed as a nucleating layer for Ag growth, compatible with the process of organic devices. 

In this paper, we select MoO_3_ for Ag percolation using the thermal evaporation method because MoO_3_ is also a good hole transport material which can act as a bi-functional layer in the designed OSCs. In some reports [42,43,44], the MoO_3_/Ag (hereafter, called MA) transparent electrodes were capped with an additional MoO_3_ layer (hereafter, the triple layer structure is called MAM) for further reducing the reflection of light at the visible range based on the principle of admittance match [45,46]. However, this is not always necessary, depending on the working wavelength range of real applications. It has been noticed that different research groups have reported quite different device performances of MA based electrodes. In 2015, Lee et al. fabricated an MAM electrode with each layer 30 nm/11 nm/24 nm thick which had a peak transmittance of 75% at 500 nm while the transmittance rapidly dropped to ~50% at 760 nm [47]. In 2016, Travkin et al. reported another MAM electrode with each layer 10 nm/10 nm/12 nm thick, showing a transmittance of 90% at 380 nm; but the transmittance slump at long wavelength range was much more severe, with the transmittance decreasing to only ~35% at 760 nm [16]. In contrast, Xu et al. reported an MA composite film with each layer 10 nm/10 nm thick which displayed relatively good transmittance at long wavelength range (~60% at 760 nm) [48]. The deviation between these results could be ascribed to their different deposition rates of Ag. In this paper, the MA electrodes fabricated at different deposition rates were compared systematically on the aspects of the transmission spectrum, surface resistance, and surface morphology. Our study indicates that with the presence of a 1 nm thick MoO_3_ nucleation layer, the Ag film of only 7 nm thick can achieve percolation and the film is porous instead of forming isolated islands. In other words, the 10 nm thick MoO_3_ layer utilized in most of the literature is not compulsory for Ag percolation. It is concluded that the increase of the deposition rate can yield obvious improvement of the surface morphology of the Ag film. The MA electrode with a 9 nm thick Ag layer shows a surface resistance of about 20 ohm/sq, and its average transmittance in the visible light range between 400 nm and 760 nm reaches 74.22%. Particularly, our optimal MA electrode displays a negligible transmittance drop at long wavelength range with the transmittance maintained at 70% at 760 nm, superior to all reported results in the literature. Furthermore, we apply the optimal MA transparent electrodes in semitransparent OSCs. 

## 2. Materials and Methods

### 2.1. Fabrication of the Transparent Electrode

Prior to beginning the fabrication process, the glasses were cleaned in an ultrasonic cleaner successively with deionized water, acetone, and isopropyl alcohol for 10 min in each round, and then they were dried in a vacuum oven at 100 °C for 10 min. Both MoO_3_ and Ag were deposited using a thermal evaporator under a base pressure below 8 × 10^−4^ Pa, and the thicknesses of the films were monitored during deposition. The deposition rate of MoO_3_ was fixed at 0.01 nm/s and the thickness of MoO_3_ as 1 nm. The deposition rates of the Ag films were varied from 0.1 to 0.9 nm/s and the thickness of Ag film was also tuned to yield the optimal optical and electrical performance at the same time.

### 2.2. Fabrication of Semitransparent Organic Solar Cell Devices

The fabricated semitransparent OSC has a configuration of ITO/ZnO/PTB7:PC_70_BM/MoO_3_/Ag. The donor PTB7 (Poly{4,8-bis[(2-ethylhexyl)oxy]benzo[1,2-b:4,5-b′]dithiophene-2,6-diyl-alt-3-fluoro-2-[(2-ethylhexyl)carbonyl]thieno[3,4-b]thiophene-4,6-diyl}) and the acceptor PC_70_BM (phenyl-C_70_-butyric-acid-methyl-ester) were purchased from 1-Material. The active layer solution was prepared by dissolving PTB7 and PC_70_BM in chlorobenzene with 3% DIO at concentrations of 10 mg/mL and 15 mg/mL, respectively. PTB7 and PC_70_BM were completely dissolved after the solution mixture was stirred vigorously at 60 °C for 12 h but the stirring needs to be maintained until use. ITO glasses were scrubbed with detergent first, and then cleaned by deionized water, acetone, ethanol, and isopropyl alcohol, successively, in an ultrasonic cleaner. After the clean ITO substrate was transferred into the glove box, the electron transporting layer of ZnO and the active layer of PTB7:PC_70_BM were made consecutively by the spin-coating method both with a speed of 1000 rpm. The realized thicknesses of ZnO and PTB7:PC_70_BM were around 30 and 100 nm, respectively. Later, the samples were placed in the chamber of the thermal evaporator. After the base pressure reached 8 × 10^−4^ Pa, the depositions of the hole transporting layer MoO_3_ and the ultrathin Ag electrode were carried out successively. The deposition rate of MoO_3_ was 0.01 nm/s, and that of Ag was 0.7 nm/s. Here, for good hole transporting property, the thickness of the MoO_3_ layer was tuned to 2 nm while the optimal thickness of the Ag film (7 nm) obtained in the step of making transparent electrodes was adopted in the OSC devices. In this paper, the active area of each device is 0.04 cm^2^.

### 2.3. Measurement

Transmittance properties of the Ag based transparent electrodes and the semitransparent OSC were measured by a commercially available angle-resolved reflection/transmission spectroscopy system (R1, IdeaOptics Instruments, Shanghai, China). The surface resistance of the fabricated electrodes was characterized by a four point probe system (Loresta AX MCP-T370, Mitsubishi Chemical Analytech, Kanagawa, Japan). Scanning electron microscopy (SEM) images of the surface of ultrathin Ag films were recorded using a thermally assisted field emission SEM system (LEO 1530, Carl Zeiss NTS GmbH, Oberkochen, Germany). Current density-voltage curve characteristics and External Quantum Efficiency (EQE) spectra of the device were also analyzed. The current density-voltage characteristics of OSCs under dark and AM 1.5 G solar illumination provided by a solar simulator (Sun 3000, ABET, Milford, CT, USA, calibrated using a standard silicon cell) were measured using the Source Meter of Keithley 2400 (Keithley, Solon, OH, USA). The wavelength dependent external quantum efficiencies of the OSCs were recorded using a CSC1011 (ZOLIX instruments, Beijing, China) which was equipped with a short arc xenon lamp source (UXL-553, Ushio, Cypress, CA, USA).

## 3. Results and Discussion

**Figure 1:**
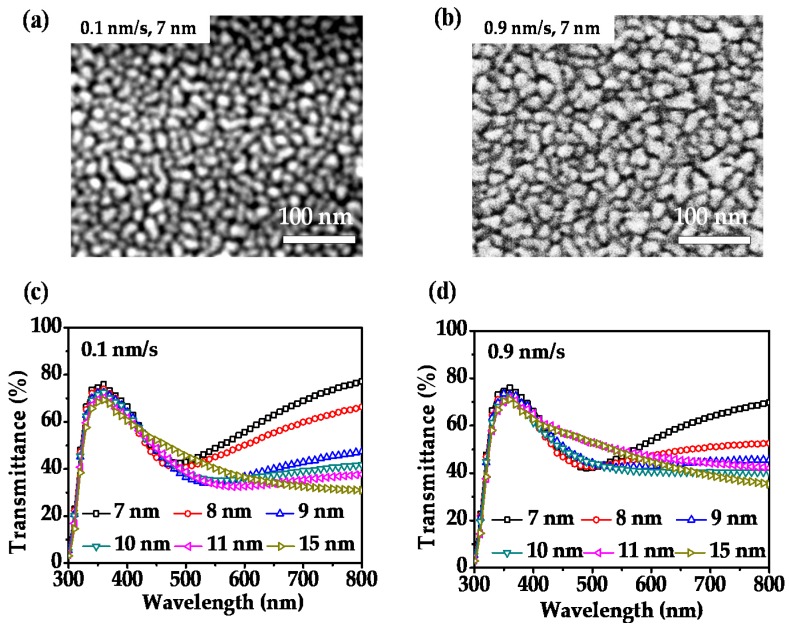
Scanning electron microscope (SEM) images of 7 nm thick Ag thin films directly prepared on glass substrate at the deposition rates of 0.1 nm/s (**a**) and 0.9 nm/s (**b**). Transmission spectra of Ag films with varied thicknesses prepared at the deposition rates of 0.1 nm/s (**c**) and 0.9 nm/s (**d**).

Before the fabrication and characterization of Ag electrodes in the form of MA, studies related to the Ag electrodes without any nucleation layer were carried out first for comparison. Figure 1a,b show the SEM images of the Ag thin films of 7 nm which were directly prepared on clean glass substrates at a deposition rate of 0.1 and 0.9 nm/s, respectively. It is known that without the nucleation layer, the growth of the Ag films on glass substrate begins with island-like particles following the Volmer-Weber mode [49,50], which can be clearly reflected from both SEM images in Figure 1a,b. No matter that the deposition rate of Ag is low or high, one can see from the SEM images that island-shaped particles are formed by directly depositing Ag on glass. By comparison, one also sees that the average grain size of Ag islands obtained under a high deposition rate of 0.9 nm/s is larger than that obtained with a low deposition rate of 0.1 nm/s, indicating that the high deposition rate is able to suppress the island growth and thereby facilitate percolation. In other words, the Ag growth forms a percolating layer more easily with a high deposition rate. This can be further verified through the characterization of transmittance properties of Ag films with varied thicknesses. Here, Figure 1c,d show the transmittance spectra of Ag films of 7 nm, 8 nm, 9 nm, 10 nm, 11 nm, and 15 nm thick with the deposition rate of 0.1 and 0.9 nm/s, respectively. It can be seen that in Figure 1c, for the low deposition rate case, the transmittance dip due to surface plasmon resonance of Ag nanoparticles always takes place when the Ag thickness is equal or below 11 nm, and the surface plasmon induced dip bears a red shift with the increase of the film thickness, ascribed to the increase of the average grain size. When the Ag thickness increases to 15 nm, the transmittance dip disappears, and a smooth spectrum is produced, reflecting that a continuous film which can conduct electrons (with a measured surface resistance of 34.02 ohm/sq) is formed. While in Figure 1d, for the high deposition rate case, the transmittance dip due to surface plasmon resonance only takes place when the Ag thickness is below 11 nm. The Ag films with thickness of both 11 and 15 nm do not show any transmittance dip in the visible range, indicating that the conductive film starts being formed at a thickness of 11 nm (with a measured surface resistance of 71.39 ohm/sq) at a deposition rate of 0.9 nm/s, which is 4 nm smaller than that at the deposition rate of 0.1 nm/s. The measured surface resistance of the 15 nm thick Ag films under 0.9 nm/s is also greatly reduced (10.26 ohm/sq) compared with the case of 0.1 nm/s deposition rate. Moreover, due to better percolation, the transmittance efficiencies for the continuous films in Figure 1d are higher than that in Figure 1c. However, even under a high deposition rate, the transmittances at the long wavelength range of the 11 nm thick Ag film are still quite low (~42% at 760 nm), similar to the reported results [16].

**Figure 2:**
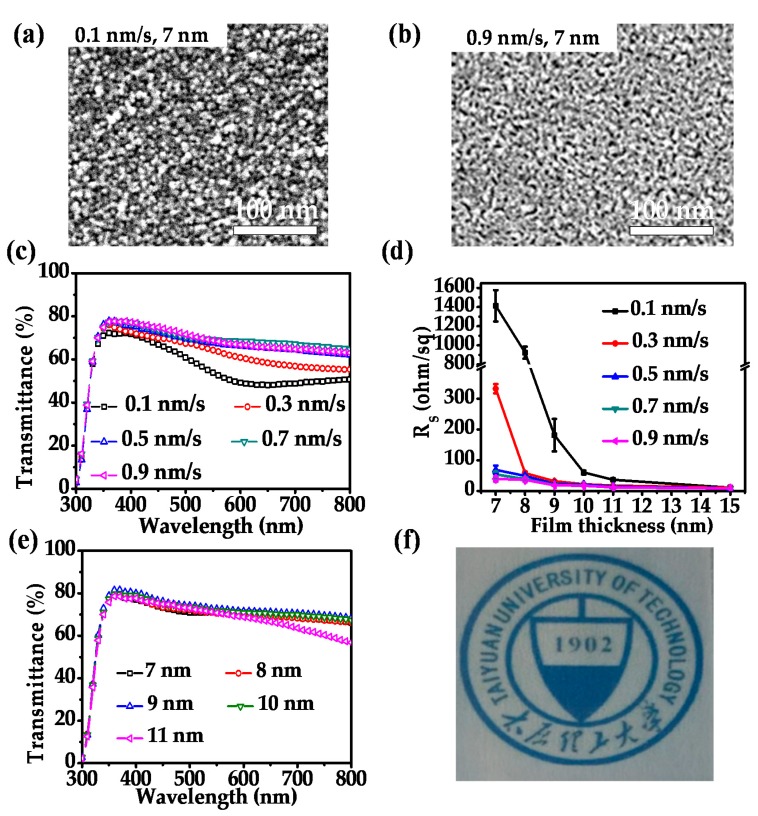
SEM images of MoO_3_/Ag (MA) films prepared at the deposition rates of 0.1 nm/s (**a**) and 0.9 nm/s (**b**); (**c**) Transmission spectra of 7 nm Ag thin films deposited on MoO_3_ nucleation layer at the rates of 0.1, 0.3, 0.5, 0.7, and 0.9 nm/s, respectively; (**d**) Surface resistances of MA films with varied Ag thicknesses which were deposited at different rates; (**e**) Transmission spectra of MA films with varied Ag thicknesses at the deposition rate of 0.7 nm/s; (**f**) Photograph of the 9 nm MA transparent electrode placed over an image.

Next, the study on ultrathin Ag film growth was carried out with the MoO_3_ nucleation layer. Here, different from most of the literature which used a MoO_3_ layer with a thickness of 10 nm or so, we found that 1 nm thick MoO_3_ is sufficiently good for functioning as the nucleation layer. Figure 2a,b show the SEM images of the Ag thin films of 7 nm which were prepared on a 1 nm thick MoO_3_ coated glass substrates (i.e., the MA films) at a deposition rate of 0.1 and 0.9 nm/s, respectively. It is observed that under the deposition rate of either 0.1 or 0.9 nm/s, that the SEM images of 7 nm thick Ag samples with a nucleation layer as shown in Figure 2a,b do not own clear island boundaries as those observed in Figure 1a,b. This means that the growth of Ag no longer follows the Volmer-Weber mode. Instead, most parts of the Ag surface are connected with some isolated black domains (corresponding to pores of the film) witnessed in Figure 2a,b. This is because the transition metal oxide MoO_3_ more easily provides oxygen bonds to Ag atoms than the insulator medium SiO_2_ [51,52], resulting in better adhesion of the Ag atoms on the MoO_3_ layer. Besides, the SEM images displayed in Figure 2a,b also deviate from each other. It was found that a 0.1 nm/s deposition rate tends to produce a continuous Ag film with some tiny bumps as reflected from the change of color in Figure 2a. In comparison, the deposition rate of 0.9 nm/s yields a quasi-flat surface; as seen there is less obvious color change taking place in Figure 2b than in Figure 2a. Such observation tells us that the process of high deposition rate lowers the selectivity of the Ag atoms toward the target substrate during growth. 

We further characterized the transmittance spectra of MA films when the Ag layer is fixed at 7 nm and its deposition rate is tuned from 0.1 to 0.9 nm/s, as shown in Figure 2c. Later, the MA film with the Ag layer of X nm is called X nm MA film. It can be seen that under a deposition rate of 0.1 nm/s, there is a very tiny dip occurring in the transmittance spectrum, and at higher rates the transmittance spectra are all smooth, displaying no dips over the investigated wavelength range. The tiny dip for the 0.1 nm/s case could be ascribed to the surface plasmon resonance produced by the bump-like geometries on the Ag surface as observed from the SEM image in Figure 2a. The transmittance efficiencies over the whole range are more or less the same for the cases of 0.5 nm/s, 0.7 nm/s, and 0.9 nm/s, which are a bit higher than those obtained under the lower deposition rate of 0.3 nm/s. Compared with the results of the thinnest continuous Ag film obtained under 0.9 nm/s without any nucleation layer (that is of 11 nm thick) as shown in Figure 1d, it was found that the participation of the 1 nm thick MoO_3_ layer enables a thinner Ag layer to become continuous (7 nm) and at the same time lifts its transmission in the visible range much higher (~68% at 760 nm). The high transmission of the MA film is because the Ag layer is porous, allowing more incoming light to transmit through compared with a perfect continuous film without any pores. 

The surface resistance of the above five different MA films were also characterized by the four point probe system as shown in Figure 2d. It was found that the conductivity property of the 7 nm MA film was improved significantly with the increase of the deposition rate. The improvement of the surface resistance of 7 nm MA film from 0.1 to 0.3 nm/s achieves around 1000 ohm/sq and the improvement from 0.3 to 0.5 nm/s is also quite high (~260 ohm/sq). The 7 nm MA films prepared at a deposition rate no smaller than 0.3 nm/s show surface resistance below 100 ohm/sq, and the higher the deposition rate, the lower the surface resistance. It was found that the lowest surface resistance of a 7 nm MA film is 40 ohm/sq, realized at the Ag deposition rate of 0.9 nm/s. 

A series of studies was then carried out for MA films with the thickness of the Ag layer varying from 7 to 15 nm. In Figure 2d, the surface resistances of all different samples are displayed. Similar to the situation taking place for 7 nm MA films, the increase of the deposition rate causes a decrease of the surface resistance for all other MA films except the 15 nm thick cases. Moreover, the decrease of surface resistance with the increase of deposition rate becomes less apparent for thicker MA films. In detail, surface resistances of 9 nm MA films do not show obvious differences between the cases of 0.7 and 0.9 nm/s and both surface resistances are around 20 ohm/sq. The 10 nm (or 11 nm) MA films perform almost the same on the aspect of conductivity when the deposition rate is no smaller than 0.3 nm/s and their surface resistances are about 17 ohm/sq (or 12 ohm/sq). It was also seen that the surface resistances between the 15 nm MA films fabricated at different rates are almost identical and their surface resistances are extremely small (around 10 ohm/sq). From Figure 2d, one can easily draw a conclusion that depositing the Ag layer with a rate above 0.5 nm/s is favorable for improving the electrical property of the transparent electrodes. Combined with the conclusion drawn from Figure 2c, we know that such a choice yields improvement of both the optical and electrical properties of the MA transparent electrodes. 

We also characterized the transmittance spectra of all the MA electrodes studied in Figure 2d (not shown). For the thickness dependent transmittance spectra of the MA electrodes, we show the situation of 0.7 nm/s rate as an example in Figure 2e because it is seen from Figure 2c that this rate produces 7 nm MA films with more or less the same spectra with respect to the rates of 0.5 and 0.9 nm/s. It was found that the performance of an 11 nm MA film is inferior to the other four cases. Table 1 shows the calculated *T_a_* and the measured *R_s_* of the MA films with different thicknesses fabricated with 0.7 nm/s. It is clearly seen that the 9 nm MA film exhibits the highest average transmittance of 74.22%; but its *R_s_* is 19.68 ohm/sq, a bit higher than the 11 and 15 nm MA films. Figure 2f is the photograph of our 9 nm MA transparent electrode placed over a paper with the logo of our university, which exhibits quite good transparency. Though the 11 nm MA film has the lowest transmittance among the five samples, its surface resistance is the best (only 12.18 ohm/sq). In applications of optoelectronic devices, the exact thickness of the Ag layer of the MA electrode should be determined case by case because the device configuration, the working wavelength range, and the semiconductor materials are varied. The ultimate decision is made based on the device performances. 

**Figure 3:**
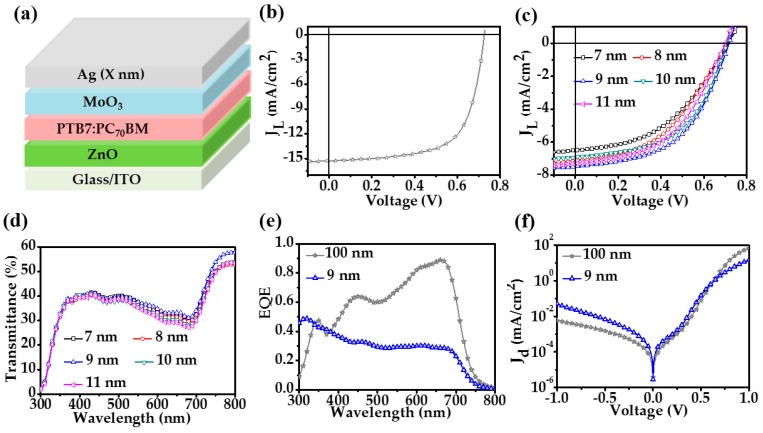
(**a**) Structure of the semitransparent organic solar cells (OSCs); (**b**) Curve of current density versus voltage for the control opaque OSC; (**c**,**d**) Curves of current density versus voltage and transmittance spectra for the MA electrode based semitransparent OSCs devices with varied Ag thicknesses when light is illuminated from the Ag electrode side; (**e**,**f**) External quantum efficiency (EQE) spectra and curves of current density versus voltage under dark for the one 9 nm MA electrode based semitransparent OSC and the control.

Finally, we applied the MA transparent electrodes in semitransparent OSCs. The device configuration is ITO/ZnO/PTB7:PC_70_BM/MoO_3_/Ag with the thickness of the top Ag layer varying from 7 to 11 nm. The device structure is shown in Figure 3a, with X (nm) denoting the thickness of the thin Ag layer. An opaque OSC which has the same configuration as the semitransparent OSCs but with the top Ag layer of 100 nm thick was first demonstrated as the control. The fabricated control opaque OSC, with the current density versus voltage curve shown in Figure 3b, exhibits a performance with PCE of 7.32% and the corresponding open circuit voltage (*V_oc_*), short circuit current (*J_sc_*), fill factor (FF), series resistance (*R_s_*), and shunt resistance (*R_sh_*) are 0.73 V, 15.31 mA/cm^2^, 66.00%, 3.22 Ω·cm^2^, and 735.94 Ω·cm^2^, respectively. Figure 3c displays the current density versus voltage curves for the MA electrode based semi-transparent OSCs with their device characteristics summarized in Table 2 as well. It was found that increasing the thickness of the Ag electrode does not cause apparent change of the open circuit voltage. However, the short circuit current varies greatly with the increase of the thickness of the Ag electrode. The device with a 9 nm thick silver layer has the highest *J_sc_* of 7.47 mA/cm^2^, that is about half of that of the opaque control. The 9 nm device which performs the best is mainly because this electrode has the highest average transmittance as shown in Table 1, which can also be seen from the transmittance spectra of different MA based OSCs as shown in Figure 3d. As can be seen, the semitransparent OSC with a 9 nm thick silver layer indeed shows the highest transmittances over the investigated wavelength range and its average transmittance *T_a_* over the visible range was calculated to be 38%. Ascribed mainly to such a high transmittance and corresponding insufficient photon absorption, the semitransparent OSCs have a much lower PCE (2.76% with a 9 nm thick Ag electrode) with respect to the opaque OSC which bears zero transmission, in accordance with the measurement of the external quantum efficiency (EQE) spectra as shown in Figure 3e. It can also be clearly observed from Figure 3d that, among these five semitransparent devices, the one with an 11 nm thick Ag electrode performs the poorest on the aspect of transmittance, in accordance with the results in Table 1. 

In addition, we found that compared with the control opaque OSC, the semitransparent OSCs do not show any obvious difference on shunt resistance. Figure 3f shows the comparison between curves of the current density versus voltage for one 9 nm MA electrode semitransparent OSC and the control. It was found that the reverse saturation current for the semitransparent OSC is a bit higher that of the control. Such a difference is acceptable as the standard deviation of shunt resistances are around 100 Ω·cm^2^. The almost unchanged shunt resistance between control and semitransparent OSCs indicates that the MA electrode does not induce extra surface defects and thus yields more or less the same surface recombination as the 100 nm thick electrode. We also found that the series resistances of the semitransparent OSCs are much higher than that of the control. The increase of the series resistance leads to a relatively poor fill factor of the semitransparent OSC, which would definitely cause insufficient charge extraction efficiency and further reduce the PCE of semitransparent OSCs on the premise of insufficient photon absorption. The poor *R_s_* of MA based OSCs is produced because the ultrathin silver electrode has a higher surface resistance compared with that of the 100 nm thick silver electrode.

Table 3 summarizes the performance parameters of MA (or MAM) based semitransparent OSCs reported in the literature and this work. By comparison, we concluded that the MA based semitransparent OSC demonstrated in this work is much superior to the two reported devices [42,43] which were also illuminated from the Ag side. Compared with the reported semitransparent OSC illuminated from the ITO side [44], the PCE of our semitransparent OSC is lower but our average transmittance is higher than twice that in the reference.

## 4. Conclusions

In this work, we systematically studied the effect of the deposition rate of Ag on the performances of ultrathin MoO_3_/Ag transparent electrodes based on the thermal evaporation method. It was found that the Ag film fabricated on top of the glass substrate shows minor improvement on film growth with the increase of deposition rate. At a deposition rate of 0.9 nm/s, the Ag film on glass substrates still have a percolation thickness of ~11 nm. In contrast, with the help of a 1 nm thick MoO_3_ nucleation layer, under a deposition rate of 0.7 nm/s, the MA film with Ag layer of 7 nm can already conduct electrons, corresponding to a surface resistance of ~54 ohm/sq. It was demonstrated that the MA electrode with the Ag layer of 9 nm fabricated at 0.7 nm/s deposition rate owns the highest average transmittance of light over the visible range (~74.22%) compared to MA electrodes with a Ag layer of other thicknesses. The transmittance performance of our optimized MA electrode is superior to all reported results in the literature, which may be ascribed to the better control of the deposition rate of the Ag film realized through the tantalum boat in our experiments. This transmittance optimized sample has a surface resistance of around 20 ohm/sq. For MA electrodes with a silver layer thicker than 9 nm, the transmittance becomes slightly lower but the surface resistance can be improved. In applications of optoelectronic devices, the exact thickness of the Ag layer of the MA electrode should be determined case by case and the ultimate decision should be made based on the device performance. For example, we applied the fabricated ultrathin MoO_3_/Ag transparent electrodes in semitransparent organic solar devices. In our device, MoO_3_ acts as not only the hole transport layer but also the nucleation layer beneficial to the formation of the Ag thin film. Through optimization, we obtained a semitransparent OSC with a PCE of 2.76% and an average transmission in the visible range of 38% when light is incident from the Ag electrode side. The power conversion efficiency of the semitransparent OSCs can be further optimized by introducing a MoO_3_ capping layer on top of the Ag electrode (in other words, using MAM electrodes) by tuning the transmittance spectrum of the MA electrode matching well with the absorption of the active layer. However, this manipulation would inevitably bring about a reduction in the transmittance of light through the device, therefore compromises between the optical and electrical performances should be made if MAM is utilized. 

## Figures and Tables

**Table 1:** The average transmittance *T_a_* calculated from measured transmittance spectra in Figure 2e and the measured *R_s_* of the MoO_3_/Ag (MA) films with varied Ag thicknesses fabricated with 0.7 nm/s deposition rate.

Ag Thickness (nm)	7	8	9	10	11
***T_a_* (%)**	71.00	71.72	74.22	72.28	69.39
***R_s_* (ohm/sq)**	53.74 ± 9.16	38.71 ± 6.59	19.68 ± 1.77	17.48 ± 2.27	12.18 ± 0.74

**Table 2:** Performance parameters of the MA electrode based semitransparent OSCs when the Ag thickness is varied under AM 1.5 G illumination at 100 mW/cm^2^ from the Ag electrode side.

Device (X/nm)		*J_sc_* (mA/cm^2^)	*V_oc_* (V)	FF (%)	PCE (%)	*R_s_* (Ω·cm^2^)	*R_sh_* (Ω·cm^2^)
7	Average	6.48 ± 0.08 (6.53)	0.71 ± 0.008 (0.71)	47.75 ± 1.89 (48.00)	2.19 ± 0.07 (2.23)	33.93 ± 5.77 (32.59)	925.87 ± 121.89 (835.11)
	Best						
8	Average	6.96 ± 0.14 (7.12)	0.71 ± 0.006 (0.71)	44.33 ± 0.58 (45.00)	2.18 ± 0.07 (2.26)	39.54 ± 2.09 (40.29)	669.53 ± 68.20 (675.17)
	Best						
9	Average	6.93 ± 0.40 (7.47)	0.72 ± 0.005 (0.72)	54.00 ± 2.45 (51.00)	2.71 ± 0.05 (2.76)	22.56 ± 3.07 (23.42)	999.04 ± 131.60 (873.40)
	Best						
10	Average	6.71± 0.16 (6.85)	0.71 ± 0.010 (0.71)	53.17 ± 0.75 (54.00)	2.54 ± 0.09 (2.63)	22.40± 2.21 (21.68)	859.18 ± 102.82 (883.63)
	Best						
11	Average	7.17 ± 0.17 (7.11)	0.71 ± 0.010 (0.72)	47.50 ± 2.38 (49.00)	2.40 ± 0.12 (2.51)	37.01 ± 6.54 (42.8)	722.22 ± 137.24 (883.82)
	Best						

**Table 3:** Performance parameters of MA (or MAM) based semitransparent OSCs reported in the literature and this work. * means the illumination is from the ITO side in that work.

MA(M) Data		Performance of Semitransparent OSCs					Reference
MA(M) Thickness (nm)	*T* at 760 nm (%)	*T_a_* (%)	*J_sc_* (mA/cm^2^)	*V_oc_* (V)	FF (%)	PCE (%)	
1/10/20	65.0	NA	2.72	0.57	61.6	0.96	[42]
6/10/40	~40.0	NA	4.64	0.54	55.0	1.39	[43]
6/7/40	NA	18.3	14.7	0.68	46.3	4.7	[44] *
2/9	70.0	38.0	7.47	0.72	51.0	2.76	This work
